# Income, Self-Rated Health, and Morbidity. A Systematic Review of Longitudinal Studies

**DOI:** 10.3390/ijerph16162884

**Published:** 2019-08-12

**Authors:** Elena Reche, Hans-Helmut König, André Hajek

**Affiliations:** Department of Health Economics and Health Services Research, University Medical Center Hamburg-Eppendorf, 20246 Hamburg, Germany

**Keywords:** income, health, morbidity, chronic diseases, chronic conditions

## Abstract

If people were asked whether income changes influence self-rated health and morbidity, they would probably answer yes. Indeed, previous studies validated this assumption, but most of them used cross-sectional data and only considered self-rated health as the decisive factor. On the other hand, there are a few studies using longitudinal data, which found a much smaller association between income and self-rated health. In order to give a conclusive overview of the current study situation, this review summarizes and examines studies which use only longitudinal data. In addition to self-rated health, the effects of income on the objective factor of morbidity were also investigated. The review includes a total of 14 papers that use data from seven different countries. It concludes that there is a small, statistically significant, positive impact of increased income on self-rated health, but a negative association between income growth and morbidity. Taking the limitations of confounders, attrition, and selection bias into account, the results may even be insignificant.

## 1. Introduction

From a macrosocial point of view, health is an important factor contributing to longevity, successful aging, or satisfaction with life. Therefore, there is a widespread interest in finding possible influencing factors and their interactions with health to gain a better understanding of the determinants of health.

Income is generally regarded as such a factor. Most people would intuitively claim that higher income improves health. Indeed, research shows clear evidence for a statistically significant, positive relationship between income and health. However, most studies use only cross-sectional data to examine this relationship [1,2,3]. Analyses using longitudinal data are rare, although longitudinal data is considered to have more explanatory power than cross-sectional data [4]. The few studies using longitudinal data found that the positive relationship is smaller than the relationship found in cross-sectional studies [5,6,7]. These analyses were conducted using longitudinal data that had been collected for at least twenty years, starting with data collected from The first National Health and Nutrition Examination Survey (1971–1975) [8]. Irrespective of this research, scientists still do not agree on a clear causal relationship between income and health [9].

A systematic review from 2011 investigated the relationship between change in income and change in self-rated health (SRH) using only longitudinal data. It was found that only a small number of studies met the inclusion criteria, as the number of existing studies with longitudinal data was small. They concluded that there is a statistically significant, small, and positive relationship between income and SRH, and that once controlled for any bias, the result would no longer be found [9].

Since the research was carried out in January 2010, these results are now almost 10 years old. The aim of this review therefore is to include the research done in the last 10 years to bring the scientific findings concerning the relationship between income and health up to date. A further aspect was added by also investigating an objective factor, morbidity, to obtain holistic view on the relation between income and health, although there are even fewer studies on this subject. This systematic narrative review analyzed studies with longitudinal data in order to find some evidence for the relationship between income change and SRH and morbidity, asking the question: “What effect does a change in household income have on SRH and morbidity when examining longitudinal data?”. To be more precise, the effect of income change on a subjective measurement parameter for general health and the effect on the objective outcome morbidity was examined. Furthermore, the effect of exogenous positive income shocks in the form of lottery winnings or the German reunification was analyzed. 

## 2. Materials and Methods

### 2.1. Inclusion Criteria

The criteria for being included in this review were use of:

1. Longitudinal data to investigate the relationship between income and health over time;
2. income in an individual/absolute form, rather than as a measure of income inequality;
3. health as:
  - SRH or a similar measurement parameter for subjective general health OR
  - several, but at least three, different chronic conditions for the variable of morbidity; and
4. a wide age range with only adults included. The age of the working population (15–65 years old) was considered the best cohort.

### 2.2. Search Strategy

The search was started in September 2018 with the parameters of income, SRH, morbidity, and longitudinal data. The variables SRH and morbidity were linked with “OR”, while income and longitudinal data were linked with “AND”. Furthermore, SRH and morbidity were linked to the other parameters via “AND”. This combination resulted in the identification of 285 articles via the PubMed database. After scanning the titles and abstracts, 272 papers were excluded for not meeting the inclusion criteria. The remaining 13 papers were analyzed by reading the whole text. Seven papers were excluded following this, with a total of six papers included in this review. Another search was carried out in March 2019 with the exact same parameters and combinations. It resulted in one additional paper being included in this review.

One of the aforementioned papers is a systematic review [9]. This paper was subject to deeper analysis to find further papers that meet the inclusion criteria. These papers were found in PubMed or Google Scholar. After scanning the papers’ title, abstract and the text, a further five papers were included in this review.

The last step of the search strategy was to analyze the references of the included papers. This analysis identified another two papers for inclusion in this study.

In total, 14 papers met the inclusion criteria and provided the basis for the subsequent analysis.

**Figure 1:**
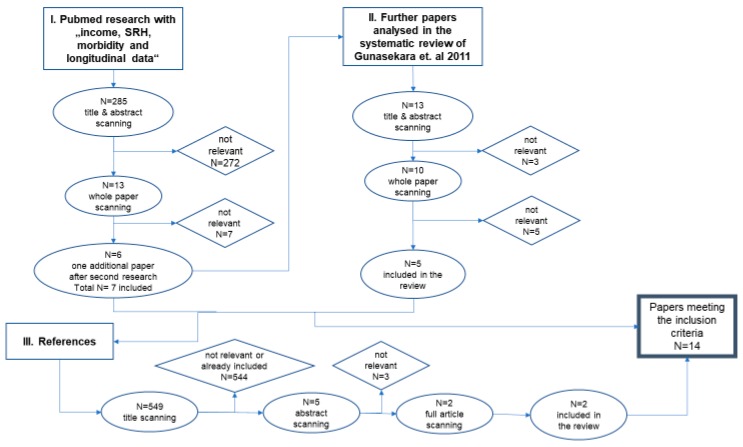
The search strategy.

Figure 1 shows the search strategy used to research the relevant papers for this review. 

### 2.3. Dependent and Independent Variable

Both dependent and independent variables were defined in different ways in many of the papers, but some papers had some definitions in common. One independent and two dependent variables are defined below.

#### 2.3.1. Income

The independent variable income was often defined as individual household income per annum. This includes employment earnings, government benefits, pensions, investments, and interests [7]. Equivalized income was also used, which takes into account the size of the household and its composition, including the number of dependent children [6,10]. In addition, the gross household income was used by almost every paper, with just two papers using the net household income [4,11]. Another exception is that two papers used lottery winnings as exogenous positive income shocks to define a change in income [12,13].

#### 2.3.2. Self-Rated Health (SRH)

In 11 of 14 papers, SRH was based on the answer to the question “In general would you say your health is excellent, very good, good, fair, or poor?” The other papers use different approaches. One paper did not include any measure of self-rated health [14], while another paper gave just three possible answers to the abovementioned question [11]. One paper asked for the number of poor mental health symptoms [13].

#### 2.3.3. Morbidity

The other possible outcome of morbidity was included in only four papers and they all defined it in different ways. One study combined 48 health symptoms to the Standardized Index of Bad Health [13]. Another paper defined the variable according to different organ systems [12], while one paper assessed the presence of specific chronic diseases using a list of 13 preselected diseases [14]. Last but not least, Fiscella and Franks considered physical examination and rated the severity of the condition in three grades [8].

### 2.4. Variations between the Papers

As described above in “Dependent and Independent Variables”, income as well as SRH and morbidity were defined in different ways. In particular, morbidity was defined differently in each of the four papers.

The time of measurement also varies across the papers with dates between 1967 and 2015. The papers analyzed a total of nine different panel survey data sets from seven countries. Furthermore, the statistical methods differ to some extent. A regression model was used by every paper except one [15]. Fixed effects regression models, which were used by only half of the studies seems to be the best method to account for time-invariant and time-varying variables [10,16].

Table 1 gives an overview of the variables, time of measurement, survey data, countries and statistical methods used in each individual paper. 

Looking at all these variations, it is not possible to conduct a meta-analysis. The scope of this paper is therefore limited to a narrative review.

## 3. Results

In total, 14 papers met the inclusion criteria and could be included in the review (Table 1).

The included papers analyzed survey data from seven different countries (Australia, China, Germany, Great Britain, New Zealand, Sweden, and the United States). In the following section of this paper, the findings will be presented according to country, alongside summaries of the panel surveys used in each the papers to ensure better comparability. Following this, the systematic review of Imlach Gunasekara et al., which included studies from four different countries and five different panel surveys, is considered separately. 

### 3.1. China

One paper analyzed the Chinese population with the help of the National Health Services Survey (NHSS) and China Health and Retirement Longitudinal Study (CHARLS).

The NHSS started in 1993 and is conducted every five years, and includes questions about socioeconomic characteristics, health status, satisfaction rate and health insurance, among other things. In total, the survey contains data from about 300,000 adults (>15 years) from 93,600 households [14].

The CHARLS began in 2011 and is repeated every two years covering topics like socioeconomic status and chronic diseases. The information was collected from 17,000 older adults (>45 years) living in 10,000 households [14].

The paper dealt with the relationship between socioeconomic status and morbidity in China. For the analysis, data was collected from the NHSS in 1998, 2003 and 2008 and from the CHARLS in the first four waves (2011–2015). The NHSS and the CHARLS allow a statement to be made as to whether the relationship exists both in the all-age cohort and in the older only cohort. The authors analyzed three morbidity outcomes (two-week incidence rate, the prevalence of chronic diseases, and the number of sick days per thousand people) and two factors of socioeconomic status (income and education) using individual fixed effect regression models and individual random effect models. It was found that the relationship between income per capita and morbidity is quadratic negative and non-linear. Furthermore, morbidity decreases by growing per capita income until a turning-point level is reached, whereby morbidity begins to increase with continuing income growth [14].

### 3.2. Germany

There is just one paper analyzing the German population employing the German Socio-Economic Panel (GSOEP). 

The GSOEP is a survey which used a sample of initially 12,000 West German adults (>17 years) in 1984. 4400 adults of East Germany were included in the sample from 1990. The data has been collected every year since then [9].

The authors examined the effect of income change on health satisfaction in both West- and East Germany after the reunification in 1990. They used data collected in the years 1984 to 2002. Adding a fixed-effects ordinal estimator and a causal decomposition technique to account for attrition bias, the results show a small, significant and positive effect of income change on health satisfaction in only East German males. While there is no significance for East German females, a small, significant effect was found in West German males and females. Furthermore, a downward trend in health satisfaction for both East and West was found by analyzing the data through 13 years since reunification [6].

### 3.3. Great Britain

Three papers included in this review looked at Great Britain. The British Household Panel Survey (BHPS) was used by all these studies to investigate the possible relationship between income and health.

This longitudinal survey started in 1991 with a sample of about 10,000 adult people (>16 years) from about 5500 households. It was continued with annual interviews covering several topics like individual and household demographics, mental and physical health, labor-force status, employment. In more recent waves, the sample size has grown to 16,000 people from 9000 households [10].

The first study examined the association between household income, income inequality, relative income and self-reported health using the first 12 waves (1991–2004) of the BHPS. In total, the paper applied three differently ordered probit regression techniques resulting in supporting the absolute income hypothesis. In other words, the absolute household income had a significantly positive effect on SRH. On the other hand, no evidence was found to support either the relative income hypothesis or the income inequality hypothesis [4].

The second paper collected data from the first 11 waves starting in 1991 to investigate the relationships between income, relative deprivation and SRH. The use of parametric and semiparametric panel data models allowed the authors to account for misspecification and heterogeneity. It was found that household income affects self-assessed health in a small but statistically significant, positive way. It also showed that large changes in income lead to small changes in SRH. The correlation was characterized as non-linear [15].

The third and last paper using the BHPS data estimated the effect of lottery prizes as a proxy for exogenous income shock on four different health outcomes (general health status, mental health, physical health problems, and health behaviors). The collected information included health data from 1996 through 2008 (wave 6–18), lottery prizes data from 1997 until 2008 (wave 7–18) and they were analyzed using three different individual fixed effects regression models. The results show statistically insignificant coefficients for the lottery prizes. To be more precise, no positive relationship between exogenous income shocks and self-rated health or physical health problems was found [12].

### 3.4. New Zealand

Altogether, three papers investigated the effect of income on health in citizens of New Zealand and all of them used the NZ Survey of Family, Income and Employment (SoFIE).

The SoFIE is a nationally representative panel survey of the resident population for a period of eight years (October 2002–September 2010). The annual interviews included adults (>15 years) from a total of 11,500 households. About 22,000 people interviewed in the first wave were followed over time. Additionally, the number of residing children in each household was identified (about 7000 at wave 1) [17].

The first paper focused on the relationship between income change and SRH and how this relationship is modified by baseline health or poverty. The methods included data from four waves of the survey (2002–2005) and a fixed-effects ordinal logistic regression model. Analyzing the variables showed a small, positive, but non-significant association between annual household income and SRH. To be more precise, an increase in income of $10,000 in one year improved the chance of reporting better SRH by 1%. The only significant modification of this relationship was caused by poor baseline health, while poverty or deprivation had no influence [7].

The second study collected information from seven waves (2002–2009) of the survey to investigate the effect of In-Work Tax Credit (IWTC) interventions on SRH. The variables of eligibility for IWTC and the amount of IWTC and SRH were analyzed using fixed effects regression analyses, allowing for the consideration of time-invariant and measured time-varying confounders. The findings showed that becoming eligible for IWTC as well as a great increase in the IWTC amount caused no difference in SRH. In addition, it was found that an increase by $1000 in the IWTC amount improved SRH by only 0.003 units, which means there is no significance in the relationship [10].

The last paper used the exact same sample with the same methods and similar variables to find out whether an unconditional tax credit for families (FTC) affects the SRH. Using eligibility for FTC and the amount of FTC as measurement parameters, the analysis resulted in a small and insignificant change in SRH in becoming eligible for FTC or receiving a $1000 increase in the FTC amount. In other words, there is no substantial effect of unconditional tax credit for families on SRH. To summarize this second and last study, it can be said that there is no difference between the effect of unconditional and employment-conditional tax-credit on SRH [16].

### 3.5. Sweden

The Swedish population was examined by two different papers using the Swedish Level of Living Surveys (LNU). 

The surveys investigated a random sample of adult population (15–75 years) starting in 1968 and repeated in 1974, 1981, 1991, 2000, and 2010. The interviews included questions with regard to health, education, and working conditions and combined the answers to register information of household income [18].

The first paper analyzed the effect of positive income shocks measured as monetary lottery prizes on health and mortality. Poisson as well as Ordinary Least Squares regression were used on the collected data from waves in 1968, 1974, and 1981. It found out that increased income causes better health. A lottery win of about SEK 100,000 in a 13-year period improves SRH by 3 percent. In addition, there is a negative association between income shocks and poor mental health, cardiovascular diseases, and headaches [13].

The second study examined income changes over time and their relationship with SRH. The longitudinal income data has been collected from the income register of 1995 to 2000 and was connected to the cross-sectional data on self-rated health of the LNU in 2000. Using logistic regression models, the findings show that decreases in absolute income have a stronger effect on SRH than income gains do. The loss of income causes adverse effects on health while an increase in income shows heterogeneous results. In summary, the study found that income instability in both ways leads to inimical effects on health [11].

### 3.6. USA

The three papers studying the residents of the USA used three different panel surveys. 

The first study investigated the effect of income on health with the help of the first National Health and Nutrition Examination Survey (NHANES) and Epidemiologic Follow-up Study (NHEFS). The first survey was conducted from 1971 to 1975 starting with a sample size of 14,407 adults (25–75 years) [19]. The Epidemiologic Follow-up Study was conducted in 1982 through 1984, 1986, and 1987, collecting mortality data [20].

The aim of the paper was to examine the associations between individual income, income inequality, SRH, morbidity, and mortality. In addition to the collected baseline data from the first National Health and Nutrition Examination Survey, follow-up data including data about SRH from 1982 through 1984 were also used. Using cumulative logistic regression models, there was a much stronger correlation between individual income and biomedical morbidity than was the case with income inequality. Also, the same connection to baseline and follow-up SRH was found [8].

The second paper used the Panel Study of Income Dynamics (PSID), which started in 1968 with nearly 5000 households. Every year, the study collected information about income and employment experiences until 1997. Data has been collected every two years thereafter. SRH-data was only gathered between 1984 and 1997 [9].

This paper focused on the impact of income variations on SRH using health data from 1984–1997 and labor income data from 1978–1994. After individual-specific fixed-effects regression analysis was carried out, the results show that positive income shocks improve health and at the same time adverse income shocks cause the opposite effect. In addition, health is improved by moving from the bottom or the top of the income distribution to the middle. The turning point between improvement and deterioration of health was found to be the 75%-percentile of income distribution [21].

The most recent paper looked at a specific part of the population using the Mexican American Cohort Study (MACS). Mexican Americans (>18 years) were recruited between July 2001 and April 2004. The information covered, among other things, demographic characteristics, medical history, level of acculturation, and social habits [22].

The study investigated the prediction of SRH before and after imputation of the missing variable yearly household income. Data was collected between 2002 and 2005 and four different methods were used to impute the missing variable income. Using a logistic regression analysis, it was found that the variable yearly household income is a good predictor for SRH. To be more precise, an increasing income of $5000 improves the odds of SRH as good or better by 11%. On the other hand, people with a good or better SRH have a significantly greater income than those with a fair or poor SRH [23].

### 3.7. Systematic Review by Imlach Gunasekara et al

The review analyzed 13 studies, which used five different longitudinal panel surveys altogether. The BHPS, GSEOP, as well as the PSID have already been summarized (see above). The remaining surveys concern the US and Australia and are called the Health and Retirement Study and the Household, Income and Labour Dynamics in Australia (HILDA). 

The Health and Retirement Study is a biennial study that started with 22,000 adults from the US (>50 years) in 1992. The focus of this study was the employment and health transition in older people [9].

The HILDA survey began in 2001 with 7683 households including nearly 14,000 individuals (>18 years) from all over Australia [9].

The aim of the review was to find the possible relationship between change in income and change in SRH in adults. All the studies used in the review were conducted either in Great Britain, USA, Germany or Australia. Most studies found a positive, small, and statistically significant association between income and self-rated health. Three of these studies found no significant associations among women. After consideration of unmeasured confounders and measurement error, the association was no longer found [9].

## 4. Discussion

Before starting the research, we expected the relationship between income and self-rated health to be significant, namely that income would have a clear, positive effect on self-rated health as shown in several cross-sectional studies before [24]. We thought that greater income would reduce stress levels and improve general life satisfaction due to greater income security (e.g., maintenance; rent) and the ability to afford more leisure activities. This improved satisfaction would increase the chance of better SRH-ratings. We also assumed that the effect of income on morbidity would be smaller, but also significant. On the one hand, there are chronic diseases with causes beyond the influence of external factors such as income. On the other hand, some of the most widespread diseases are cardiovascular diseases and diabetes, which are mostly caused by bad health behaviors, which in turn can be influenced by income. The hypothesis was that higher income leads to individuals investing more in healthy nutrition and sports activities, so that these diseases do not occur.

In summary, most of the analyzed papers found clear evidence for a significant, positive relationship between income and both SRH and morbidity. Surprisingly, this association was very small and would likely be rendered insignificant, once adjusting for confounders and bias. It was even more surprising that two studies characterized the relationship as non-linear and presented a specific turning-point. Reaching this turning point, the studies suggest that SRH or morbidity starts to worsen while income continues to grow [14,21].

### 4.1. The Relationship between Income and Self-Rated Health

In total, there are nine studies and one systematic review examining the effect of income on SRH or some similar variables for self-rated health. Therefore, this issue has been most frequently explored in the 14 analyzed papers. To sum up, five studies found a significant, positive association between income and SRH, but only a very small one. Two of these papers only covered a short time period of three or four years, while the other three covered a longer period of 11 or 13 years. Having similar results, the duration of the measurements seems to be irrelevant for the results. At this point, it would be interesting to find out whether this irrelevance is also present when investigating much longer time periods as for example a whole life. There is currently no such data available. 

Four studies found either no significance or inconsistent results in relation to the effect of income on SRH. Two of them examined employment-conditional or unconditional tax-credits as income shocks and found a small but insignificant association between income shocks and SRH [10,16]. This association can be distorted by selection bias due to different losses of participants according to ethnicity and education and therefore reduce the effect [10]. The results of another paper are consistent with these two by finding a small positive but statistically insignificant association [7]. However, the authors failed to account for attrition bias due to only including individuals who responded in all waves of the survey [7]. The last study found that decreases in income or income loss have a greater influence on SRH than an increase in income [11]. Given the fact that income loss often goes hand in hand with the loss of job, the greater impact of income loss on health can be explained by the occurrence of income insecurity. Nevertheless, the significance of this study should be questioned, since it linked cross-sectional data to longitudinal data, which is not as powerful as surveys using only longitudinal data [11].

The other papers presenting a significant positive association between income and SRH also include factors that limit their explanatory power. The most common problem of these studies were unmeasured confounders. The income–health association is quite complex due to many variables which can possibly modify this interaction. Some studies ignored important confounders such as employment status, social support and family structure [4,8].

The aim of all papers was to investigate the pathway from income to health in order to analyze the relationship between income and health. Therefore, the occurrence of the opposite pathway (from health to income) called reverse causality was mentioned as another limitation [15,25]. 

Selection bias should also be mentioned. Two papers stated this bias as their limitation. One study included a greater number of women in its sample (87%). Consequently, these findings may not be generalizable to the whole population [23]. Accounting for all these limitations may reduce the significance of the results.

In summary, the results of the studies are the same as in the analyzed systematic review of Imlach Gunasekara et al. Most of the studies in the analyzed systematic review as well as in this review showed a small, statistically significant, and positive relationship between income and SRH [9]. This review identified evidence for a non-linear characteristic in the relationship between income and SRH, and one paper even identified a specific turning point [21]. To be more precise, with improving income, SRH increased initially until a particular turning point, where SRH then started to decline [21]. 

### 4.2. The Relationship between Income and Morbidity

Only two studies investigated the relationship between income and morbidity. The reason for this may be that the common variable SRH considers all aspects of health. Therefore, morbidity is also included in measures of SRH. Nevertheless, SRH and morbidity should be distinguished, because SRH is a subjective variable whilst morbidity can be examined objectively. Both studies found a significant negative association between income and morbidities [8,14].

Due to very different ways of measuring morbidity, it is quite complicated to compare these studies with each other. One study used biomedical morbidity measured through physical examination and classified according to the severity of the condition [8]. The other paper collected data on the prevalence of chronic diseases [14]. However, the study only collected data on 13 kinds of chronic diseases, excluding a number of chronic diseases [14]. These diseases may be differently affected by income and so the result may be biased. To put it more clearly, these missing diseases could be affected in other ways by increasing income than the investigated 13 diseases were. Therefore, the results of the study may not be significant, or a positive relationship may even be found.

The main difference between this study and almost all the other studies is the definition of income. Mostly, the other studies, in particular the paper analyzing the relationship between income and morbidity, used household income, while this study used income per capita [14]. This additional difference between the two studies makes comparability even more difficult.

However, in previous literature, the negative association between income and morbidity was also found in Europe and the USA [26,27]. On the other hand, there was no significant relationship found in Canada [28]. These studies from Europe, the USA and Canada are cross-sectional or examined only a small age group, so they do not meet the given inclusion criteria of this paper.

In summary, the two analyzed papers do not present new evidence, but they can confirm the results of previous literature.

### 4.3. The Effect of Exogenous Positive Income Shocks

There are three studies examining a special form of income change. They investigated the effect of exogenous positive income shocks on SRH and morbidities either in the form of lottery winnings or as the result of the German reunification.

Two studies using lottery prizes as income shocks showed inconsistent results. One paper found statistically significant association between these income shocks and SRH as well as morbidity [13], while the other found no evidence for a positive relationship [12].

There are some differences between the two studies, which may explain the conflicting results. Firstly, the analyzed surveys come from different countries (Sweden and Britain) and the sample size was different. The small sample in the Swedish survey of about 3000 adults may be less meaningful than the British sample of about 16,000 people. Secondly, analyzing the Swedish data, the authors defined their outcome differently from the British study by using the number of poor mental health symptoms. Interestingly, the results of the British study also found a significant positive effect on mental health. In this case, the results are consistent, only leaving a difference in the effect of income shocks on morbidity [12,13].

There is a major advantage, but also disadvantage, in using lottery winnings as a proxy for income changes. On the one hand, lottery winnings as exogenous income shocks are better suited to investigate the causality of the relationship due to the unexpectedness of income growth. On the other hand, the generalizability is questionable, since only those willing to take risks are included in the sample [12].

The analysis of the German reunification showed a significantly positive effect of income change on self-assessed health satisfaction for males only. The authors also found a downward trend of health satisfaction for both East and West over a 13-year period after reunification, suggesting the positive effect is a short-term effect. The main strengths of the study cover the exogeneity of reunification and the use of fixed-effects models. The exogeneity allows a greater chance to examine the causality between income and health and, due to the fixed-effects models, they are able to account for individual heterogeneity. However, only 26% of East Germans were interviewed in all waves, which means that the sample has suffered a large decrease in the number of participants over time (panel attrition) [6].

All three studies are forms of natural experiments collecting data at least over 10 years.

Apart from the strengths of such natural experiments in terms of determining the causality of the relationship between income and health, one major problem should also be considered. Namely, the health outcomes of these studies may not be comparable to those studies that presuppose a permanent increase in income. The same individual may be affected in different ways in cases of an exogenous income shock or permanent income change [9]. Thus, it may play a role whether the income was the result of hard work or pure chance. 

## 5. Limitations

First, there were only a few papers which met the inclusion criteria and could be included in the review. In contrast to the large number of available cross-sectional analyses, there are still few studies using only longitudinal data on this topic. The advantage of using longitudinal data is to be able to consider any unobservable individual effects, which may be important disruptive factors for the relationship between income and health [4]. 

The review mostly considered the same dependent and independent variables, same age span, and at least similar sample sizes. Nevertheless, the differences between short-term and long-term observations and between fixed-effects and non-fixed effects in the reviewed studies cause significantly diverging results [29]. This lack of robustness suggests that the association between income and health is more complicated than previously assumed [29]. 

Looking at the countries from which the data was analyzed, it is noticeable that these are exclusively industrialized countries. The results for developing countries may differ from those for industrial countries due to the different living conditions. It therefore can be hypothesized that the living conditions of people from industrialized countries with a certain wealth might not be as strongly influenced by an increase in income as might be the case with people from, for example, developing countries. There are therefore many research opportunities in this area. Since only one database (PubMed) was used for research, possible suitable studies from other databases were not considered.

Another source of error could have occurred during the search for suitable studies. When scanning titles and abstracts, papers might have been excluded erroneously from the review due to poorly worded abstracts [9]. 

Finally, publication bias is a problem of every systematic review. There is always the possibility that analyses remain unpublished and obviously cannot be found [9]. 

## 6. Conclusions

In total, 14 studies with data from seven different countries and 11 panel surveys were analyzed in this review. 

The results did not meet our expectations. Overall, the majority of the papers did indeed find a small, but significant positive effect of income change on some measurement parameter for general self-rated health. In addition, the association between income and morbidity was found to be significantly negative. That means that growing income reduces morbidity. However, accounting for all the disruptive factors as for example family structure and employment status, reverse causation and selection bias, these results may not be significant. This means that the initial expectations were only fulfilled to a limited extent. There is a positive relationship between income and self-rated health, but it is unexpectedly small.

Looking at

◦ the multitude of possible disruptive factors,
◦ the presumably different responses to exogenous income shocks and permanent income increases, and
◦ the different research methods producing various results, it becomes clear that the association between income and health is very complex and needs further examination.

Because there could be only a few papers included in the review and longitudinal surveys are superior to cross-sectional analyses, there should be more research done with longitudinal data. Particularly, there is a lack of literature using long-term observations over 13 years or data from developing countries. There is the possibility that studies from low-income countries may found a different characterization of the relationship between income and health. More work is therefore needed, especially in low to middle-income countries.

Moreover, to date the relationship between income and morbidity has not been sufficiently investigated. Since this objective measurement parameter probably causes less recall bias than SRH, further research should also be done in this area.

Intuitively, one may assume that income changes have a big effect on self-rated health and morbidity. Most people tend to assume that an increase in income automatically means an increase in health as well. Personal health and public policy decisions may be influenced by this assumption.

However, and quite unexpectedly, the findings of our study suggest that there is only a hardly recognizable positive impact of increased income on health. The relationship between income and health seems to be more complex than generally assumed. This indicates that decisions based on the previously mentioned assumption might not be as substantiated as thought before.

## Figures and Tables

**Table 1:** Summary of included papers in the review.

Author & Year	Country	Sample Size	Age	Percent Women	SRH (Self-Rated-Health)	Morbidity	Income	Survey & Statistical Methods	Time of Measurement	Results
Jiang et al. 2019	China	NHSS (National Health Services Survey): N = 300,000 out of 93,600 households CHARLS (China Health and Retirement Longitudinal Study): N = 17.000 out of 10.000 households	NHSS: adults 15+ CHARLS: adults 45+	N/A	N/A	Prevalence of chronic diseases →one of the 13 kinds, two-week incidence rate, the number of sick days per thousand people	Real income per capita (NHSS); average annual income per capita deflated (CHARLS)	National Health Services Survey (NHSS) and China Health and Retirement Longitudinal Study (CHARLS) data individual fixed effect regression model and individual random effect model, pooling (FGLS = feasible generalized least squares); pooling (OLS = ordinary least squares)	NHSS data from 1998, 2003, 2008 CHARLS data from wave 1–4 from 2011–2015 only using data from 2011, 2013, 2015	There is a negative quadratic relationship between income per capita and morbidities. In addition, the relationship is found to be non-linear. At first morbidity decreases with growing per capita income. However, when income per capita reaches a specific turning-point level, morbidity begins to increase with continuing income growth.
Frijters et al. 2005	Germany	East: 46,953 person year observations on N = 6198 persons West: 176,770 person year observations on N = 20,617 persons	adults 18+	50–52%	Self-assessed health satisfaction scale 1–10 self-rated health (SRH) 1–5 scale	N/A	Equivalized pre-tax household income, relative household poverty	German Socio-Economic Panel (GSOEP); fixed effects ordered logit regression model	East Germans data: 13 waves from 1990–2002 West Germans data: 19 waves from 1984–2002	There is a very small positive and statistically significant effect of large increase in real household income on the health satisfaction for East German males, but not females. A similar small effect for western males and females was also found. Studying the average health satisfaction over 13 years since reunification, a downward trend in health satisfaction for both East and West Germans was found.
Lorgelly and Lindley 2008	Great Britain	Annual N = 8645 Overall N = 71,598	adults 16+	53%	Scale 1–5; recoded in a scale 1–4 with poor and very poor combined	N/A	Net total annual household income	The British Household Panel Survey (BHPS); ordered probit regression technique; pooled ordered probit (POP), random effects ordered probit (REOP), fixed effects ordered probit (FEOP)	First 12 waves from 1991–2004	There is a significant, positive relationship between self-rated health and the absolute household income.
Jones and Wildman 2008	Great Britain	Annual N = 10,000 Overall N = 113,310	adults 16+	ca. 54%	Scale 1–5 Recoded in 1 = good or excellent and 0 = fair, poor, very poor	N/A	Equivalized and deflated annual household income	BHPS (The British Household Panel Survey); parametric and semiparametric panel data models	First 11 waves starting in 1991	There is a clear evidence of income having a significant, positive but small effect on self-assessed health. Large changes in income cause small changes in health. It is shown, that there is a non-linear relationship between income and health.
Apouey and Clark 2015	Great Britain	Annual: N = 10,000–16,000 out of 5500–9000 households Overall: N = 110,000	adults 16+	ca. 54%	Scale 1–5	Health problems with arms, legs, hands; sight; hearing; skin conditions/allergy; chest/breathing; heart/blood pressure; stomach or digestion; diabetes	Positive income shocks as lottery prizes	BHPS (The British Household Panel Survey); three models with individual fixed effects regression models	Health data from wave 6–18 from 1996–2008 lottery prizes data from wave 7–18 from 1997–2008	The coefficients on any prize are insignificant. In other words, there is no evidence of a positive correlation between exogenous income and general health. In addition, there is no significant relationships between lottery winnings and physical health problems. It was found, that there is a significant positive effect on mental health.
Imlach Gunasekara et al. 2012	New Zealand	N = 22,165 out of 11,500 households	Adults 15+; middle age ca. 41	54.5%	Scale 1–5	N/A	Household income (employment earnings, government benefits, pensions, investments and interest)	NZ Survey of Family, Income and Employment (SoFIE); fixed-effects ordinal logistic regression model	Four waves from 2002–2005	An increase in income of $10,000 over one year increased the odds of reporting better SRH by 1% (OR 1.01, 95% CI 1.00 to 1.02). Poor baseline health significantly modified the association between income and SRH while poverty or deprivation did not modify the association.
Pega et al. 2013	New Zealand	N = 6900 out of 11,500 households	Adults 15 +	56.3%	Scale 1–5	N/A	Equivalized gross total annual family income;the dollar amount of IWTC (In-Work Tax Credit)	NZ Survey of Family, Income and Employment (SoFIE); fixed effects regression analyses	Seven waves from 2002–2009	A $1000 increase in the IWTC amount increased SRH by 0.003 units (no significance). Becoming eligible for IWTC or a substantial increase in the IWTC amount was not associated with a difference in SRH over the short term.
Pega et al. 2014	New Zealand	N = 6900	Working age 19–65	56.3%	Scale 1–5	N/A	Equivalized gross total annual family income; the dollar amount of FTC (Family Tax Credit)	NZ Survey of Family, Income and Employment (SoFIE); unadjusted and fully adjusted fixed effects regression analyses	Seven waves from 2002–2009	The unconditional tax credit for families had no short-term effect on SRH. There is no difference between the impact of unconditional and employment-conditional tax-credit on SRH.
Lindahl 2003	Sweden	N = 2948	Adults middle age 53.3	56%	Number of poor mental health symptoms	48 health symptoms combined to the Standardized Index of Bad Health	Income including sources as work, capital, and government transfers	Swedish Level of Living Surveys (SLLS) Poisson as well as OLS regression	Three waves from 1968, 1974, 1981	Winning 100,000 Swedish kronor (SEK) on lotteries in a 13-year period (almost 8000 per year) increases general health by 3 percent. Income shocks are negatively associated with poor mental health, cardiovascular diseases and headaches. Furthermore, the income change reduces the chance of being overweight.
Miething and Åberg Yngwe 2014	Sweden	N = 5142 (SRH Data) N = 3377 (Income Data)	Working age 30–64	ca. 50%	Good, bad and something in between; recoded in good and poor (= bad and something in between)	N/A	Individual disposable income (income after taxation and welfare transfers) = net income	The Swedish Level of Living Survey (SLLS); the income register; logistic regression models	SRH-data from 2000 (cross sectional) income-data from 1995–2000 (longitudinal data)	Decreases in absolute income have a greater effect on self-rated health than income gains have over time. Loss of income is a threat for health whereas increases in income shows inconsistent results. Income instability in either way shows an adverse association with health.
Fiscella and Franks 2000	USA	N = 14,407	Adults 25–74	N/A	Scale 1–5	Physical examination: laboratory, EKG, pulmonary function, radiologic test results; severity of conditions: minimum, moderate, severe	Total annual family income; 12 income categories ranging from under $1000 to $25,000 and over	The first National Health and Nutrition Examination Survey and Epidemiologic Follow-up Study; cumulative logistic regression models	Three waves from 1982–1984	Individual income has a much stronger relationship with biomedical morbidity and baseline and follow-up self-rated health than income inequality has.
Halliday 2007	USA	N = 13,800	Working-aged people 30–60	ca. 53%	Scale 1–5, recoded in good = 1 or 2, bad = 4 or 5 and 3 is omitted	N/A	Individual labor income; adverse income shocks (less income or unemployment)	Panel study of income dynamics (PSID); individual-specific fixed-effects regression analyses	Health data from 1984–1997 labor income data from 1978–1994	Positive income shocks tend to improve health outcomes. Movements from the bottom and the top of the income distribution to the middle leads to better health. The transition from income below the 75%-percentile to above this percentile causes an adverse effect on health (turning point). Adverse income shocks have negative effects on self-rated health.
Ryder et al. 2011	Houston, USA	N = 4162	Middle age 42.85 ± 14.87	87.3%	Scale 1–5, recoded in 0 (fair or poor) and 1 (good, very good or excellent)	N/A	Gross annual household income; four different methods to impute missing income data	The Mexican American Cohort Study (MACS); logistic regression analysis	Four waves from 1 July. 2002–31 December 2005	The “yearly income” was a good predictor of SRH outcome. The odds of SRH as good or better increased by 11% for each $5000 increment in yearly income. People with a good or better SRH have a significantly greater yearly income than the people with fair or poor SRH.
Imlach Gunasekara et al. 2011 Systematic review	Great Britain, USA, Germany, Australia	five longitudinal surveys in 13 studies	Often adults 15+	Various	Scale 1–5	N/A	BHPS, GSEOP, HRS (Health and Retirement Study): annual equivalized household income PSID (Panel Study of Income Dynamics): change in income-to-needs ratio; labor income HILDA (Household, Income and Labour Dynamics in Australia): income less than 50% of median income = poverty	British Household Panel Survey (five studies); Panel Study of Income Dynamics (four studies); German Socioeconomic Panel (three studies); Health and Retirement Study (one study); Household, Income and Labour Dynamics in Australia (HILDA) Survey (one study)	In total from 1967–2005	Most studies (ten of 13) found that income change had a small, statistically significant positive association with SRH. Three studies out of these 10 found no significant relationship among women.

## ER/HHK/AH

Final approval of the version to be submitted.
